# Supplementary material for: The causal relationship between the human gut microbiota and pyogenic arthritis: a Mendelian randomization study
Source: Front Cell Infect Microbiol. 2024 Nov 26;14:1452480. doi: 10.3389/fcimb.2024.1452480 (PMC11629706; doi:10.3389/fcimb.2024.1452480)
Supplement: Supplementary file 1 [file DataSheet1.zip › mendelian test/ebi-a-GCST90027637.csv_forest.pdf]

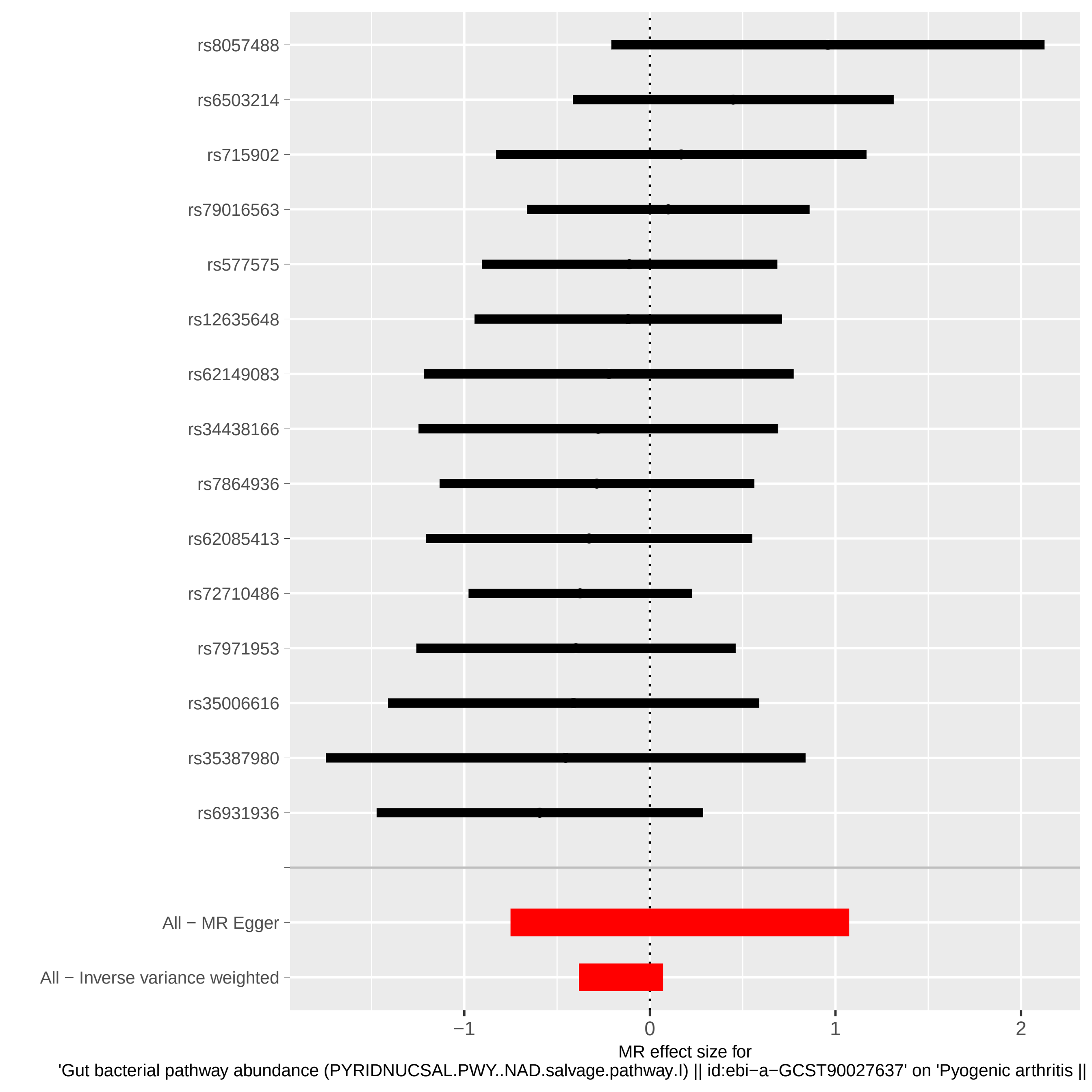
rs8057488
rs6503214
rs715902
rs79016563
rs577575
rs12635648
rs62149083
rs34438166
rs7864936
rs62085413
rs72710486
rs7971953
rs35006616
rs35387980
rs6931936
All − MR Egger
All − Inverse variance weighted
−1
0
1
2
MR effect size for
'Gut bacterial pathway abundance (PYRIDNUCSAL.PWY..NAD.salvage.pathway.I) || id:ebi−a−GCST90027637' on 'Pyogenic arthritis ||
